# A Permeable Cuticle Is Associated with the Release of Reactive Oxygen Species and Induction of Innate Immunity

**DOI:** 10.1371/journal.ppat.1002148

**Published:** 2011-07-28

**Authors:** Floriane L'Haridon, Angélique Besson-Bard, Matteo Binda, Mario Serrano, Eliane Abou-Mansour, Francine Balet, Henk-Jan Schoonbeek, Stephane Hess, Ricardo Mir, José Léon, Olivier Lamotte, Jean-Pierre Métraux

**Affiliations:** Department of Biology, University of Fribourg, Fribourg, Switzerland; Michigan State University, United States of America

## Abstract

Wounded leaves of *Arabidopsis thaliana* show transient immunity to *Botrytis cinerea*, the causal agent of grey mould. Using a fluorescent probe, histological staining and a luminol assay, we now show that reactive oxygen species (ROS), including H_2_O_2_ and O_2_^−^, are produced within minutes after wounding. ROS are formed in the absence of the enzymes Atrboh D and F and can be prevented by diphenylene iodonium (DPI) or catalase. H_2_O_2_ was shown to protect plants upon exogenous application. ROS accumulation and resistance to *B. cinerea* were abolished when wounded leaves were incubated under dry conditions, an effect that was found to depend on abscisic acid (ABA). Accordingly, ABA biosynthesis mutants (*aba2* and *aba3*) were still fully resistant under dry conditions even without wounding. Under dry conditions, wounded plants contained higher ABA levels and displayed enhanced expression of ABA-dependent and ABA-reporter genes. Mutants impaired in cutin synthesis such as *bdg* and *lacs2.3* are already known to display a high level of resistance to *B. cinerea* and were found to produce ROS even when leaves were not wounded. An increased permeability of the cuticle and enhanced ROS production were detected in *aba2* and *aba3* mutants as described for *bdg* and *lacs2.3*. Moreover, leaf surfaces treated with cutinase produced ROS and became more protected to *B. cinerea.* Thus, increased permeability of the cuticle is strongly linked with ROS formation and resistance to *B. cinerea*. The amount of oxalic acid, an inhibitor of ROS secreted by *B. cinerea* could be reduced using plants over expressing a fungal oxalate decarboxylase of *Trametes versicolor*. Infection of such plants resulted in a faster ROS accumulation and resistance to *B. cinerea* than that observed in untransformed controls, demonstrating the importance of fungal suppression of ROS formation by oxalic acid. Thus, changes in the diffusive properties of the cuticle are linked with the induction ROS and attending innate defenses.

## Author Summary

This study provides an explanation for the strong resistance to *B. cinerea* observed in wounded plants or plants with cuticular defects. We have observed that a production of ROS and a permeable cuticle is common to all these situations. ROS, that include hydrogen peroxide, are known inducers of resistance and can also act directly against the invading fungus. Degradation of the cuticle by exposure to cutinase also results in the production of ROS and resistance. These observations lead to a model where the cuticle plays a central role as a barrier against water-soluble elicitors from the surface. Under normal circumstances, the cuticle does not allow the passage of elicitors and no responses are induced. Under conditions where the cuticular barrier is broken, ROS and resistance are induced. This illustrates why plants that are in fact permanently exposed to potential elicitors do not constantly induce immune responses: this only takes place once the cuticle has been permeabilized, for example after an infection with a pathogen. This study also demonstrates how a cuticle-degrading pathogen avoids the generation of ROS by producing an effector that interferes with ROS production. Removal of this effector restores both ROS and resistance.

## Introduction

The cuticle is mainly considered as a constitutive barrier against water loss, irradiation, xenobiotics or pathogens [1], [2]. The structure of this lipid boundary layer covering aerial parts of plants is made of waxes covering and interspersed in cutin, a polymer layer formed by a network of esterified Ω-hydroxylated fatty acids that are produced and secreted by the epidermis cells [3]. Waxes comprise a mixture of very long-chain fatty acids (24–36 carbon atoms) that seem ubiquitously present in most plant species. In addition, triterpenes, β-diketones as well as phenylpropanoids are associated with the wax fraction [4], [5]. The enzymatic machinery for wax biosynthesis is in the endoplasmic reticulum and members of a subfamily of ATP binding cassette (ABC) transporters export the resulting products through the membrane to the cell wall [5]. In many plant species the cutin polyester contains C_16_ or C_18_, fatty acids as well as glycerol [6], [7]. The fatty acids in the cutin can be hydroxylated at midchains (C8, C9, or C10) or in Ω-positions and are linked together or to glycerol by ester bonds. It is still unclear if the cutin polymers exist as free polymers or if they are anchored in some ways to the cell wall [8]. The polymerization and the transport of the cutin precursors are likely to occur in the cell and conveyed to the cell wall *via* oleophilic droplets, secretion vesicles, lipid transfer proteins (LTP) or ABC transporters [7], [8]. While considerable knowledge is available on single components, the detailed chemical structure of the entire cuticle is still not known and the relation between the structure and the biological function of the individual components remains to be defined.

The cuticle has been proposed to be a physical barrier to the penetration by pathogens. This intuitive view is supported by the fact that most pathogens that penetrate directly through the cell wall produce cutinase. However, the biological relevance of cutinase or cutinolytic lipase could not be documented in all cases. Studies using antibodies directed against cutinase support a role for this enzyme in pathogenicity [9], [10]. Moreover, the effects of cutinase disruption were studied in various fungal pathogens, but only few studies found supportive evidence [11], [12], while most other results question the function of cutinase as a breaching enzyme [13]–[16]. These contradictory results might be the consequence of the functional redundancy of these enzymes, making gene disruption experiments difficult to interpret.

Components of the cuticle might function as important developmental cues perceived by invading microorganisms [6]. For instance, cutin monomers can induce the expression of cutinase [17], [18] or act as a plant signal for the induction of germination and appressorium in fungal pathogens [17]–[20]. Similarly, surface waxes can also affect the development of fungi at the plant surface [21].

The plant itself might also perceive degradation products of its own cuticle. Synthetic cutin monomers of the C18 fatty acid family applied on leaves of barley or rice increased resistance to *Erysiphe graminis* and *Magnaporthe grisea* respectively, without detectable direct fungicidal activity [22], [23]. Cell cultures of *Solanum tuberosum* respond to cutin monomers by medium alkalinization, ethylene (ET) production and accumulation of defense-related genes [23]. Abraded cucumber hypocotyls respond to cutin hydrolysates of cucumber, apple and tomato by producing H_2_O_2_ [24] that has been repeatedly associated with defense either as a signal, as an executer of cell death or as cofactor in the strengthening of the cell wall [25]–[27]. A decrease in the lesion size caused by *Rhizoctonia solani* was observed when bean leaves were inoculated with spore droplets amended with a fully active cutinase compared to droplets with an inactive cutinase or without cutinase [28]. These observations support the notion that plants have the potential to recognize breakdown products of the cuticle and activate defense-related mechanisms. Experiments with transgenic plants over expressing an active fungal cutinase in the apoplasm (CUTE plants) also support these observations [29].

An alternative explanation was provided by observations in CUTE plants as well as a series of mutants with defects in the formation of the cuticle such as *lacerata* (*lcr*; affected in the cytochrome P450-dependent enzyme CYP86A8, likely to be involved in fatty acid hydroxylation of cutin monomers) [30], *bodyguard* (*bdg*; impaired in a member of the a/b hydrolase family associated with the organization of the cutin polyester) [31] or *bre1/lacs1* (a mutant of the long-chain acyl-CoA synthetase2, LACS2, involved in the development of the cuticle and essential for its biosynthesis) [32]. All those mutants display a strong resistance to *B. cinerea*; this was always associated with an increased cuticular permeability and production of a diffusate endowed with growth-inhibiting activity against *B. cinerea* [32]. The increased cuticular permeability was proposed to allow the diffusion of toxic compound(s) from the cell to the surface or facilitate the passage of MAMPs or DAMPs (microbe or damage-associated molecular patterns [33]) from the surface to the inside of the cell, resulting in increased resistance [32], [34].

Since biochemical alterations of the cuticle were found to increase the resistance of plants to pathogens, physical alterations of the cuticle, such as wounding were tested to see if they could also increase the defense potential of the plant to virulent necrotrophic pathogens. Wounding of *Arabidopsis thaliana* leaves leads to strong and transient immunity to the virulent pathogen *B. cinerea*. This resistance is strictly limited to the wound site and is independent of the major plant defense signaling pathways involving salicylic acid (SA), jasmonic acid (JA), and ET [34].

In this work, we have attempted to further our understanding of the molecular events taking place after wounding. A rapid formation of ROS has been observed after wounding and ROS can act as a signal for innate immunity but can also serve as an oxidant for lignification [35], [36]. This prompted us to carry out observations on the formation of ROS under our conditions of wounding. A strong correlation was observed between ROS formation and resistance to *B. cinerea*. We discovered that ABA is involved in the regulation of ROS production most likely causing changes in the permeability of the cellular envelope. This led to the finding that an increase in ROS also takes place in plants where cuticular permeability was affected by mutations or simply by a digestive treatment with cutinase. We propose that the cuticle acts as a sensor for pathogens that invade directly through the cell wall, leading to ROS formation whenever the cuticle is degraded. *B. cinerea* is known to form oxalic acid that can potentially prevent ROS formation. In line with this observation we have also shown that transgenic plants constitutively expressing an oxalic acid-degrading enzyme recovered their ability to produce ROS in response to *B. cinerea* infection and were resistant to this fungus.

## Results

### Wounding induces an oxidative burst that is accompanied by resistance to *B. cinerea*

**Figure 1:**
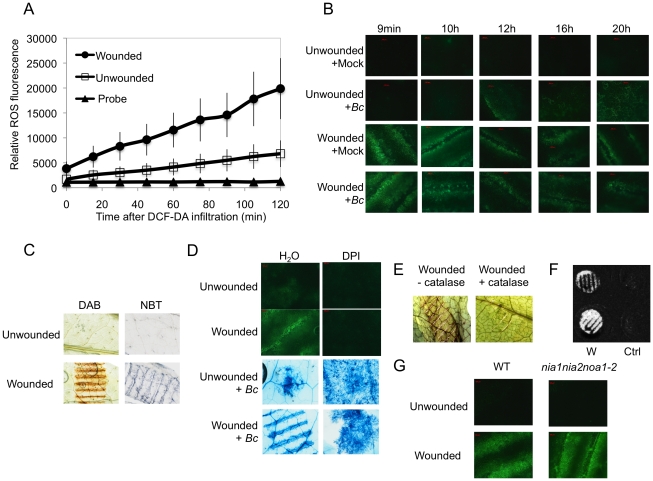
A: ROS production in response to wounding in leaves of *A. thaliana.* (A) Fluorescence after DCF-DA staining was measured on leaf discs using fluorescence spectrophotometry. The unwounded and wounded leaf discs were infiltrated with the DCF-DA probe and the fluorescence was directly measured at intervals of 15 min during 120 min (n = 12; ±SD). The experiment was carried out 3 times with similar results. (B) Time-course of ROS production observed as DCF-DA fluorescence by fluorescence microscopy in unwounded or wounded *B. cinerea* (*Bc*)-inoculated leaves compared to mock controls. After treatment, all plants were kept under humid conditions. The experiment was carried out 5 times with similar results. (C) H_2_O_2_ formation observed as DAB staining and superoxide (O_2_^−^) formation observed as NBT staining in unwounded or wounded leaves. Plants were stained immediately after wounding. The experiment was carried out 3 times with similar results. (D) Effect of DPI on ROS formation (measured as DCF-DA fluorescence) and growth of *B. cinerea* (determined by Trypan blue staining) in unwounded or wounded leaves. Leaves were treated for 24 h with DPI, then rinsed and either wounded, immediately stained and examined for fluorescence or inoculated with *B. cinerea* (*Bc*) and incubated in humid condition (symptoms were observed at 3 dpi). (E) The effect of catalase on ROS formation was tested by infiltration of catalase (1100 U ml^−1^) immediately prior to wounding. Plants were stained with DAB immediately after wounding. The experiments in Fig 1D and E were carried out 5 times with similar results. (F) Production of H_2_O_2_ in unwounded control (Ctrl) or wounded (W) leaf discs detected by the chemiluminescence reaction with luminol immediately after wounding. The experiment was carried out 3 times with similar results. (G) Production of ROS in unwounded and wounded WT plants and in *nia1nia2noa1-2*, a triple mutant deficient in nitrate reductase (NIA/NR)- and Nitric Oxide-Associated1 (AtNOA1)-mediated NO biosynthetic pathways. ROS were detected as DCF-DA fluorescence immediately after wounding. The experiment was carried out 3 times with similar results.

**Figure 2:**
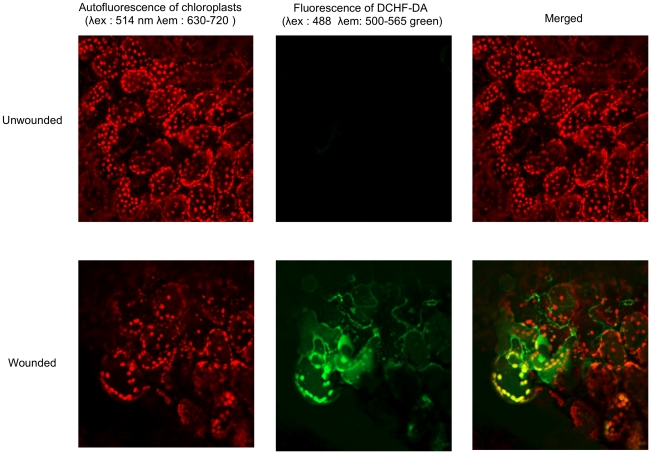
Subcellular localization of ROS at wounded sites in *A. thaliana* leaves. Leaves were infiltrated with DCF-DA, then wounded and ROS accumulation was observed by laser confocal microscopy immediately after wounding. The experiment was carried out 3 times with similar results.

Wounding *A. thaliana* leaves with forceps as previously described [34] lead to an increase of fluorescence when leaves infiltrated with the 5-(and-6)-carboxy-2,7-dichlorodihydrofluorescein diacetate (DCF-DA) probe were viewed under the microscope (Fig. 1B). This dye detects a broad range of oxidizing reagents including H_2_O_2_ and O_2_^−^ [37]. A detailed time-course of ROS production was determined in a fluorimeter using wounded leaf discs. Fluorescence appeared within the first minutes after wounding and increased steadily thereafter (Fig. 1A); unwounded controls also showed a detectable increase, likely the result of wounding inflicted during the preparation of leaf discs. In whole excised leaves, fluorescence started to be detected 2 min after wounding (Fig. S1). After inoculation with *B. cinerea*, fluorescence started to accumulate 12 h after inoculation (Fig. 1B) and wounded leaves inoculated with *B. cinerea* showed the same behavior as mock-treated wounded leaves (Fig. 1B). Staining of wounded leaves with diaminobenzidine (DAB) or with nitroblue tetrazolium (NBT) identified production of H_2_O_2_ and O_2_^−^ respectively, at the wounding site (Fig. 1C). ROS production was seriously diminished with a concomitant increase in fungal growth on leaves that were treated with DPI, an inhibitor of superoxide formation (Fig. 1D). Infiltration of leaves with catalase prevented the coloration of wounding sites with DAB confirming the production of H_2_O_2_ upon wounding (Fig. 1E). The luminol assay [38] was used to detect the formation of H_2_O_2_. A strong luminescence became visible at the wound sites (Fig. 1F). Wound-induced ROS and wound-induced resistance (WIR) to *B. cinerea* were still detected in mutants of *NADPH oxidase D* (*atrboh D*) and *F* (*atrboh F*) as well as in the double mutant (*atrboh D/F*) meaning that other genes are possibly implicated in ROS production (Fig. S2). ROS accumulation was still detected after wounding of the triple mutant (*nia1nia2noa1-2*) that is impaired in the biosynthesis of NO [39] (Fig. 1G). Laser confocal microscopy (LCM) was used to determine the localization of ROS in leaves treated with DCF-DA. In wounded leaves the fluorescence mainly localized at chloroplasts in mesophyll cells and to some extent to membranes in mesophyll and epidermal cells (Fig. 2). We have also tested the effects of H_2_O_2_ applied on leaves together with *B. cinerea.* H_2_O_2_ at concentrations as low as 1 nM caused enhanced resistance to fungal infection (Fig. S3A). This concentration is lower than the IC_50_ value of H_2_O_2_ (determined at 8.3 mM) for inhibition of hyphal growth *in vitro* (Fig. S3B). Taken together, these results show that wounding leads to a rapid burst of ROS, including H_2_O_2_, which could potentially take part in the resistance of *A. thaliana* to *B. cinerea.*

### Wounding can be disconnected from ROS and resistance in the presence of ABA

**Figure 3:**
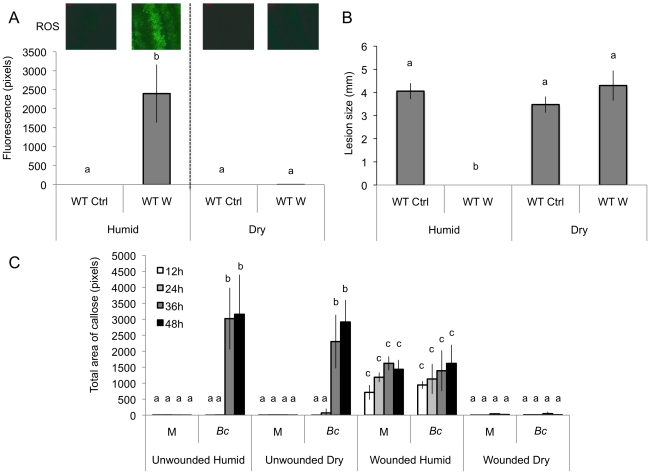
The effect of humidity on resistance to *B. cinerea*, ROS and callose accumulation after wounding. Wild type (WT) leaves were wounded and maintained for 1.5 h under high humidity in tightly covered well-watered trays (humid) or in uncovered trays at room conditions (dry) prior to ROS or infection with *B. cinerea* or callose detection. (A) Densitometric quantification of ROS production, (B) resistance to *B. cinerea*. W: wounded; Ctrl: unwounded control plants. (C) Callose formation. M: mock; *Bc*: *B. cinerea*-inoculated. For ROS production (n = 4; ±SD), one representative image of the fluorescent leaf surface was placed above each histogram as a visual illustration. For resistance (n = 48; ±SE) and callose formation (n = 4; ±SD), all plants were kept under humid conditions after treatment and the experiment was carried out twice with similar results. Different letters above each bar represent statistically significant differences (Dunn's test; P<0.05).

**Figure 4:**
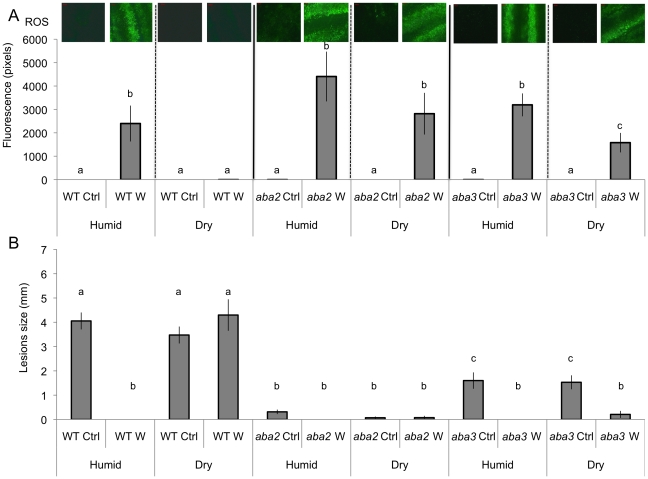
Wounding in mutants of ABA biosynthesis. Leaves were wounded and maintained for 1.5 h under high humidity in tightly covered well-watered trays (humid) or in uncovered trays at room conditions (dry) prior to ROS detection or infection with *B. cinerea*. W: wounded; Ctrl: unwounded control plants. (A) Densitometric quantification of ROS production in unwounded and wounded *aba2*, *aba3* mutants and in WT plants. For ROS production (n = 4; ±SD), one representative image of the fluorescent leaf surface was placed above each histogram as a visual illustration. Different letters above each bar represent statistically significant differences (Dunn's test; P<0.05). (B) Effects of wounding on resistance to *B. cinerea* in *aba2* and *aba3* mutants and WT plants. After *B. cinerea* inoculation, all plants were kept under humid conditions (n = 32; ±SE); the experiment was repeated twice with similar results. Different letters above each bar represent statistically significant differences (Dunn's test; P<0.05).

**Figure 5:**
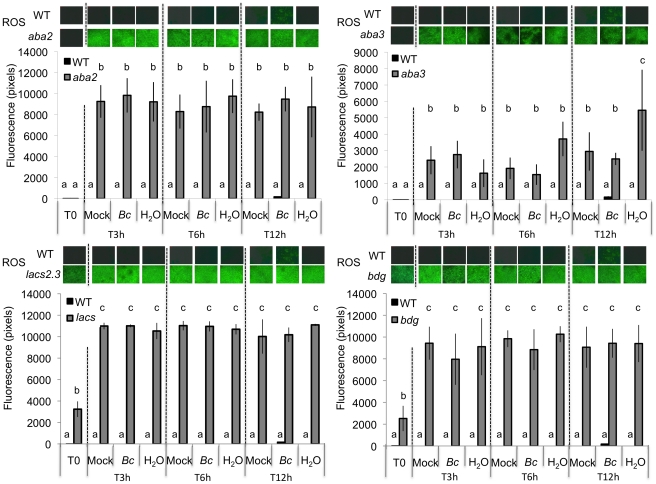
Densitometric quantification of ROS production in ABA and cuticle mutants. ROS production at 3, 6, 12 h post inoculation with *B. cinerea* (*Bc*), mock or H_2_O treatments in *aba2* and *aba3* as well as in *bdg* and *lacs2.3* mutants compared to the WT. After treatment, all plants were kept under humid conditions. Low level of fluorescence density in the WT were detected only after *B. cinerea* at 12 h post inoculation and were not detected in response to H_2_O or mock treatment. For ROS production (n = 4; ±SD), one representative image of the fluorescent leaf surface was placed above each histogram as a visual illustration. Different letters above each bar represent statistically significant differences (Dunn's test; P<0.05).

**Figure 6:**
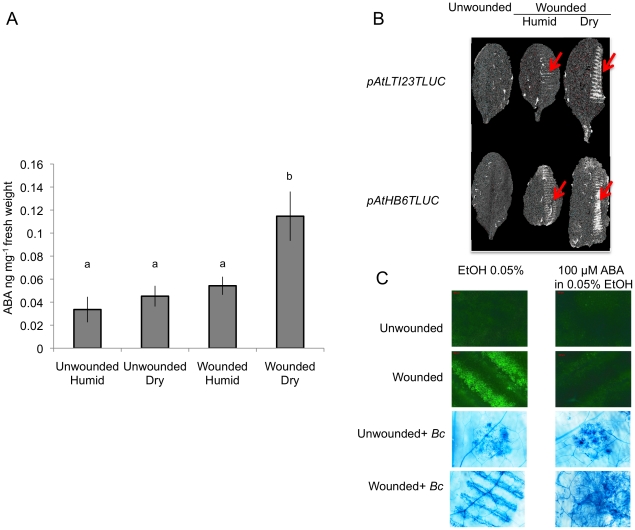
ABA accumulates after wounding under dry conditions and affects resistance to *B. cinerea*. Leaves were wounded and maintained for 1.5 h under high humidity in tightly covered well-watered trays (humid) or in uncovered trays at room conditions (dry) prior to measurement of ABA or luciferase activity. (A) Measurement of ABA in ng mg^−1^ fresh weight of plant tissue in unwounded or wounded plants, incubated under humid or dry conditions (n = 6; ±SD). Different letters above each bar represent statistically significant differences (Dunn's test; P<0.05). (B) Expression of *pAtLTI23T::LUC* or *pAtHB6T::LUC* in wounded leaves incubated either under humid or dry conditions compared to unwounded plants. The wounds inflicted by the forceps (arrows) show a stronger expression of the *LUC* gene in plants incubated under dry conditions. The experiment was repeated 3 times, one typical result is represented. (C) Effect of exogenous ABA treatment (leaf discs were floated on 100 mM ABA, 1 d prior to wounding). After wounding, ROS (measured as DCF-DA fluorescence) and growth of *B. cinerea* (*Bc*) (leaf discs were floated on water and inoculated; Trypan blue staining was carried out 2 d after inoculation) were determined (the experiment was carried out 3 times, one typical result is represented).

We observed that both ROS production and WIR could be strongly impaired when the plants were maintained uncovered for 1.5 h at ambient humidity after wounding before the measurements of ROS and resistance (Fig. 3A and B). Thereafter, we will refer to these conditions as dry in contrast to the humid incubation environment in covered trays. In contrast, ROS production and WIR to *B. cinerea* remained unaffected when, after wounding, plants were kept for 1.5 h under high humidity in covered plastic trays (Fig. 3A and B). Maintaining plants under dry conditions after wounding strongly reduced wound-induced callose formation, a typical defense reaction to wounding (Fig. 3C). The strong effects of a dry environment on the suppression of H_2_O_2_ production and WIR to *B. cinerea* made us suspect a possible involvement of ABA in the suppression of ROS. Indeed, mutants blocked in the late steps of ABA biosynthesis, such as *aba2* and *aba3*, were not blocked in ROS production after wounding under dry conditions and induced WIR in response to *B. cinerea* (Fig. 4). Both unwounded *aba2* and *aba3* mutants showed a marked resistance to *B. cinerea* accompanied by a faster and more intense ROS production after *B. cinerea* inoculation compared to WT plants (Figs. 4B and 5). The kinetics of ROS production was then also tested in unwounded *aba* mutants and showed that ROS were already released 3 h after exposure to water, PDB medium (mock treatment) or *B. cinerea* infection without any wounding (Fig. 5). The level of ABA was increased in wounded plants incubated in dry conditions (Fig. 6A). To confirm an increase in the level of ABA, the expression of ABA-dependent genes *RAB18, RDB29* and *NCED23* was tested. These genes showed an increase in expression after wounding and mock treatment as well as wounding and *B. cinerea* in dry conditions that was clearly detectable at 15 min after treatment (except NCED23 in mock-treated plants) (Fig. S4). Changes in ABA levels were further tested using transgenic plants containing a *LUC* reporter gene under the control of the ABA-specific promoters *LTI23* or *HB6*. The activity of the reporter gene was mainly observed at the wound site and it was stronger in wounded plants incubated under dry compared to humid conditions (Fig. 6B). Furthermore, exogenous applications of ABA at 100 mM led to a suppression of ROS and WIR in response to *B. cinerea* (Fig. 6C). Thus, ABA is likely to be involved in the suppression of wound-induced ROS when plants are kept under dry conditions [25].

### Common phenotypes between *aba* mutants and cuticle mutants

**Figure 7:**
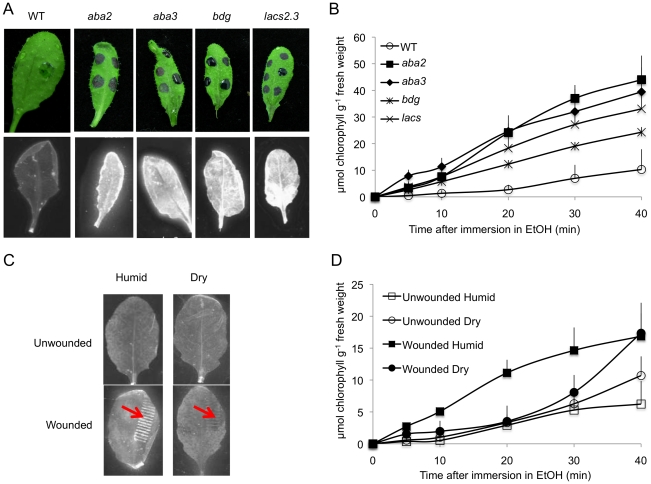
Cuticle permeability is impaired in ABA mutants and after wounding under humid conditions. Permeability of the cuticle in ABA and cuticle mutants (positive controls) or WT plants (A) and (B). (A) Upper panels: a droplet of toluidine blue was placed on the leaf surface for 2 h in high humidity then the leaf surface was rinsed with water. The blue stain that remains attached to the cell wall is indicative of a permeable cuticle. Lower panels: leaves were bleached overnight in ethanol then stained with Calcofluor white that binds to cellulose, and viewed under UV light. Calcofluor staining to the leaf is indicative of a permeabilized cuticle (all experiments were carried out 12 times, one typical result is represented). (B) Leaves were placed in ethanol and the release of chlorophyll was followed over time. Chlorophyll leached out more rapidly in all mutants compared to WT indicating a higher cuticle permeability (n = 6; ±SD). Permeability of the cuticle in unwounded and wounded plants incubated under humid or dry conditions (C) and (D). (C) Leaves were wounded and maintained for 1.5 h under high humidity in tightly covered well-watered trays (humid) or in uncovered trays at room conditions (dry). The permeability of the cuticle was assessed using Calcofluor white. Wounding (arrow) followed by incubation under humid conditions lead to white staining visible at the wound sites and to a lesser extent in other parts of the leaf. Wounding followed by incubation in dry conditions showed no staining (the experiment was carried out 12 times, one typical result is represented). (D) Chlorophyll leached out more rapidly in plants incubated under humid conditions compared to plants incubated under dry conditions (n = 5; ±SD).

**Figure 8:**
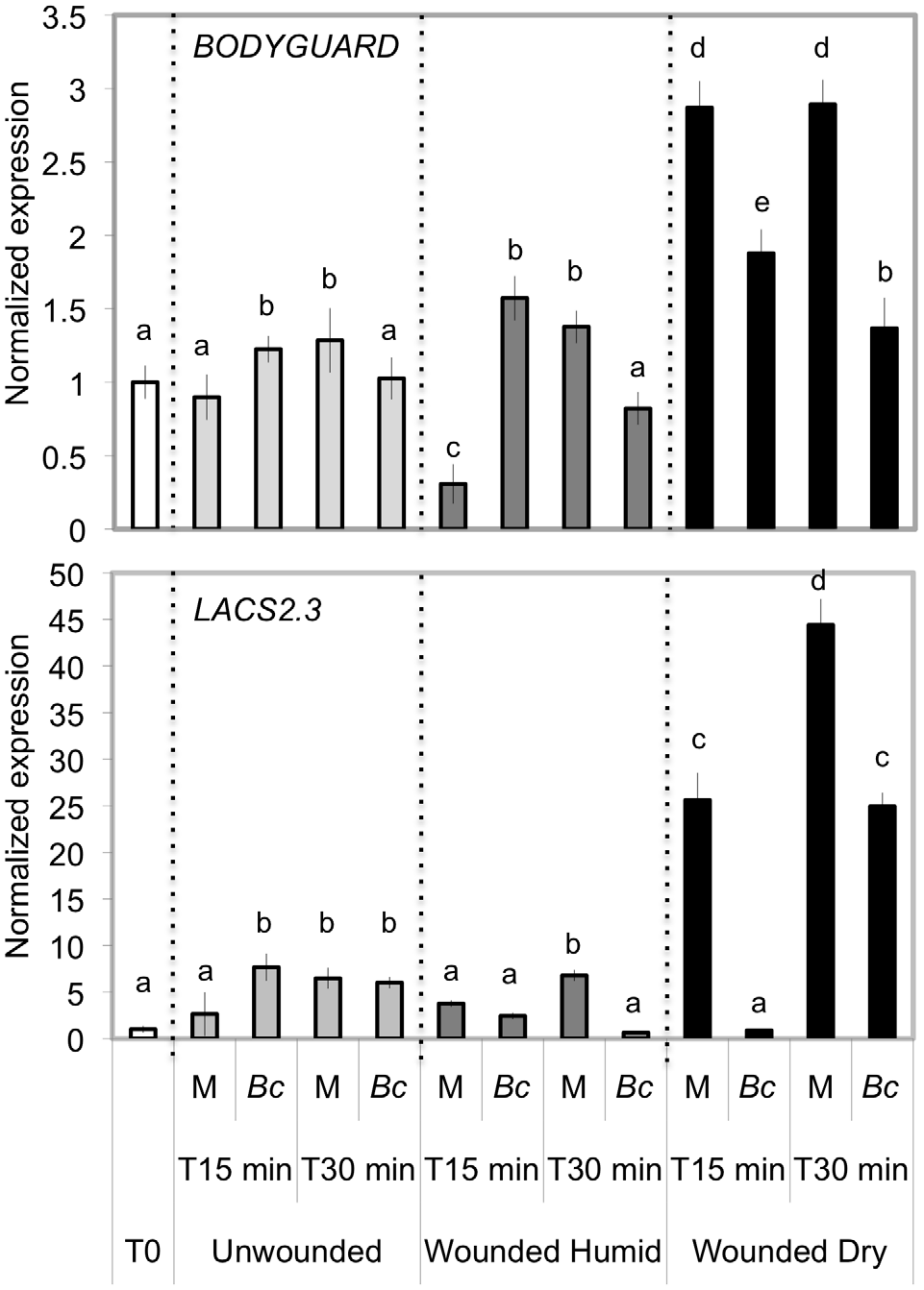
Expression of genes involved in cuticle formation in wounded and unwounded plants. Leaves were wounded and maintained for 1.5 h under high humidity in tightly covered well-watered trays (humid) or in uncovered trays at room conditions (dry) prior to expression of *BDG* and *LACS2.3* genes. Gene expression was determined 0, 15 or 30 min after wounding in plants incubated under humid or dry conditions and either mock-inoculated (M) or inoculated with *B. cinerea* (*Bc*) (n = 3; ±SD). The experiment was carried out twice with similar results. The expression levels of unwounded control plants behaved similarly under dry or humid conditions. Different letters above each bar represent statistically significant differences (Dunn's test; P<0.05).

The resistance to *B. cinerea* displayed in *aba* mutants was comparable to that observed in mutants with permeable cuticles such as *bdg* or *lacs2.3* [32]. We therefore tested and confirmed that *bdg* and *lacs2.3* also produced ROS after wounding (data not shown). Furthermore, both *bdg* and *lacs2.3* strongly displayed DCF-DA fluorescence after treatment with water, mock or *B. cinerea* compared to WT plants (Fig. 5). The cuticle of *bdg* and *lacs2.3* was previously shown to be more permeable and it was postulated that this feature would allow an easier diffusion of elicitors into the cell or an improved passage of antibiotic substances towards the surface [32]. Consequently, we tested if the *aba* mutants also display alterations in cuticle permeability. This was tested using the cell wall stain toluidine blue applied as droplets on the adaxial side of leaves as previously described [32]. Results showed that *aba2* and *aba3* mutants strongly stained in blue, as did *bdg* and *lacs2.3* compared to WT plants, indicating altered cuticular properties (Fig. 7A). We have ascertained that stomatal density did not interfere with the toluidine blue tests. The density of stomata in WT Col-0, *aba2, aba3, bdg* and *lacs2.3* mutants showed some differences that could however not account for the toluidine blue staining observed only in the mutants compared to WT plants (Fig. S5). Calcofluor staining was also used to visualize permeable cuticles [32] and marked differences were obtained between WT Col-0 and *aba2, aba3, bdg* and *lacs2.3* mutants (Fig. 7A). Increased efflux of chlorophyll is another measure of cuticular permeability [31], [40]. When dipped in ethanol, *aba2* and *aba3* as well as *lacs2.3* and to a lesser extent *bdg* mutants released chlorophyll faster than WT Col-0 plants (Fig. 7B) thus corroborating the results of the toluidine blue and Calcofluor tests. Cuticular permeability was also compared when wounded plants were incubated under humid or dry conditions. Calcofluor staining was more intense at wounded sites in plants incubated under humid conditions compared to dry conditions indicating a better access of Calcofluor to the cell wall glucans (Fig. 7C). Similarly, chlorophyll leaching proceeded more rapidly when tested on wounded leaves incubated under humid compared to dry conditions (Fig. 7D). Furthermore, the expression of the *BDG* and *LACS* genes involved in cuticular biosynthesis were compared in wounded plants incubated under humid or dry conditions. Wounding under dry conditions enhanced the expression of *BDG* or *LACS* genes, compared to wounded plants incubated under humid conditions. While *BDG* was already enhanced within 15 min after wounding and dry conditions, the expression of *LACS2.3* was clearly higher 30 min after treatment under the same condition (Fig. 8). In all experiments, expression of both genes after wounding followed by *B. cinerea* inoculation was not as extensive as the expression after wounding and mock treatment. The composition of the cuticle was altered in wounded plants after incubation in dry conditions compared to humid conditions as was the composition of the cuticle in *aba2* and *aba3* mutants compared to WT plants (Fig. S6). Furthermore, exogenous treatment of *A. thaliana* with ABA decreased the cuticular permeability (Fig. S7). ABA might therefore be involved in the control of a wound repair mechanism that decreases the permeability of the cuticle.

### Cutinase treatment leads to ROS and enhanced resistance to *B. cinerea*

**Figure 9:**
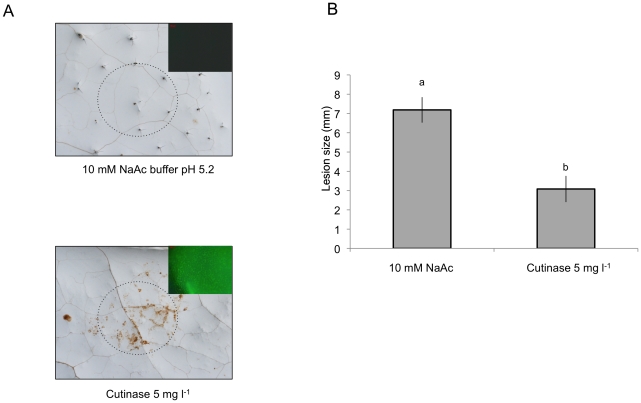
ROS production and resistance to *B. cinerea* in response to exogenous treatments with cutinase. A droplet containing cutinase (5 mg l^−1^, a non-toxic concentration; see [47]) was applied to the surface of an *A. thaliana* leaf. After 72 h in high humidity, the drop was removed, and (A) the leaf was stained with DAB or DCF-DA (insert) to detect ROS production or (B) replaced by *B. cinerea* spores (6 µL; 5×10^4^ spores ml^−1^) applied at the same location. After 72 h, lesion sizes were determined (n = 27; ±SE). The experiment was carried out twice with similar results. Different letters above each bar represent statistically significant differences (Dunn's test; P<0.05).

We have further tested the relation between cuticle, permeability, ROS and resistance to *B. cinerea* after a localized treatment with cutinase (from *Fusarium solani*, prepared using heterologous expression in *S. cerevisiae*). Localized application of cutinase led to ROS production visualized with DAB and DCF-DA staining (Fig. 9A) as well as to an increase in resistance to *B. cinerea* (Fig. 9B). These experiments support the hypothesis that plants can perceive and react to the degradation of the cuticle.

### Removal of oxalic acid, a pathogenicity factor of *B. cinerea* leads to increased ROS and resistance to *B. cinerea*

**Figure 10:**
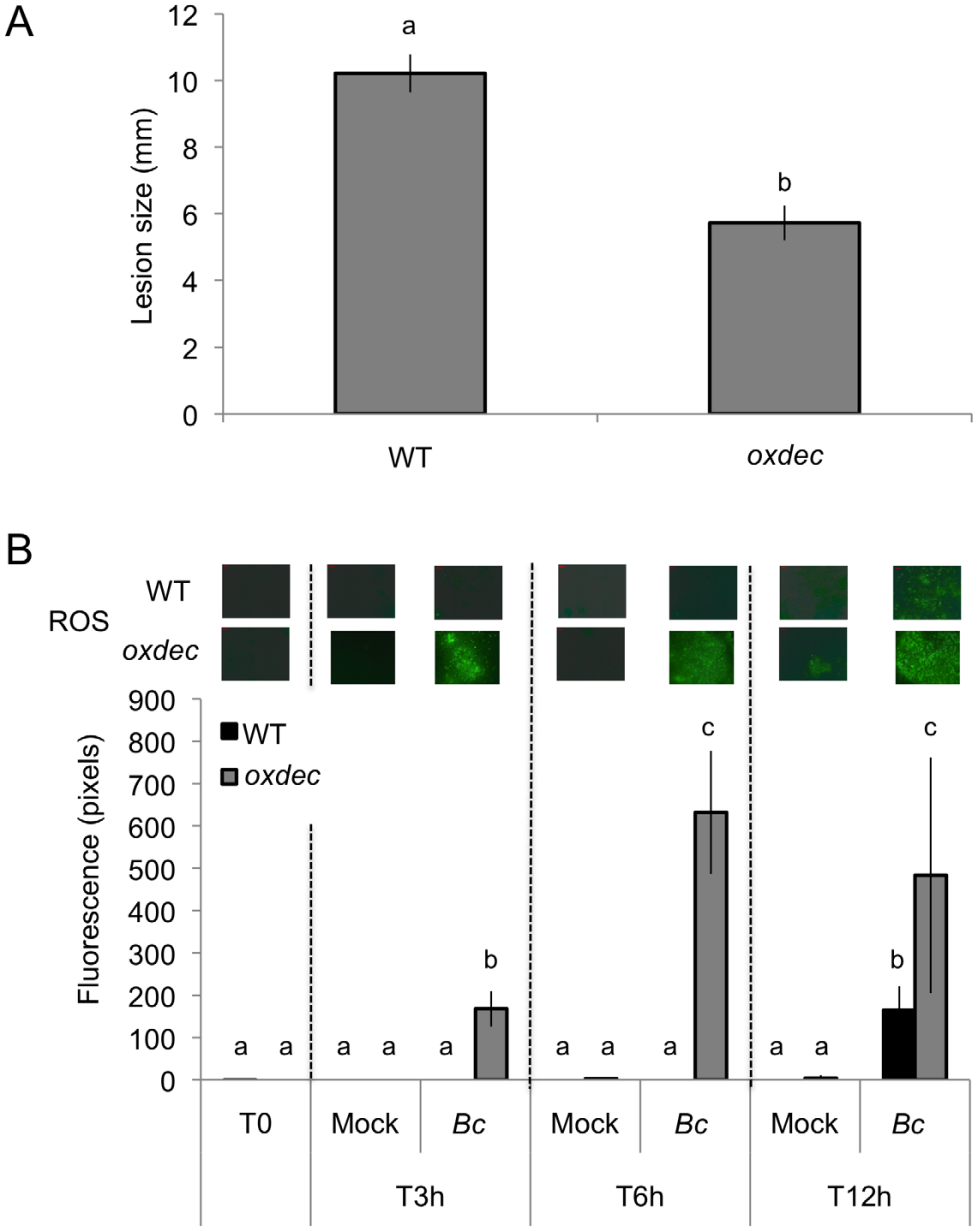
Effect of *OXALATE DECRABOXYLASE* over expression in *A. thaliana* on resistance to *B. cinerea* and ROS production. (A) Resistance displayed by T3 *A. thaliana* lines over expressing the *OXALATE DECARBOXLYLASE* gene from *T. versicolor* (*oxdec* plants); all plants were previously checked for the over expression of the oxalate decarboxylase activity (n = 130; ±SE). Different letters above each bar represent statistically significant differences (Dunn's test; P<0.05). (B) Densitometric quantification of ROS production at 3, 6, 12 h post inoculation with *B. cinerea* (*Bc*) and mock in *oxdec* plants compared to the WT. After treatment, all plants were kept under humid conditions. Low level of fluorescence density in the WT is detected only after *B. cinerea* at 12 h post inoculation and not in the mock treatment. For ROS production (n = 4; ±SD), one representative image of the fluorescent leaf surface was placed above each histogram as a visual illustration. Different letters above each bar represent statistically significant differences (Dunn's test; P<0.05).

The cutinase produced by *B. cinerea* during infection [16] might potentially cause ROS production during the early stages of infection. But the data presented in Figs. 1B and 5 show an increase in fluorescence beyond 12 h after inoculation. *B. cinerea* was reported to release oxalic acid during infection [41]. Oxalic acid inhibits ROS production in tobacco and soybean cells [42] and might potentially slow down ROS production during the infection. The importance of oxalic acid as a suppressor of ROS was tested using transformed *A. thaliana* over-expressing an oxalate decarboxylase gene from the basidiomycete *Trametes versicolor* [43]. By removing oxalic acid released by *B. cinerea*, we would predict an increase in both ROS formation and resistance. In line with our expectations, transgenic T3 lines that over expressed the *OXALATE DECARBOXYLASE* gene and exhibited increased oxalate decarboxylase activity showed an increase in resistance to *B. cinerea* (Fig. 10A). ROS appeared as early as 3 hours post inoculation at sites inoculated with *B. cinerea* (Fig. 10B) in transgenic plants compared to controls. Thus, oxalic acid produced by *B. cinerea* might help the fungus to avoid the effects of ROS produced during the early steps of infection.

## Discussion

We have previously reported a very marked resistance in *A. thaliana* to *B. cinerea* in response to localized wounding. This resistance is based on priming of camalexin synthesis, of the expression *GLUTATHIONE S TRANSFERASE 1* (*GST1*) gene, and MAPK kinase activity [34]. Here, we have followed up these observations and described early events associated with WIR. We have described the production of ROS within 2 minutes at the site of wounding using the fluorescent dye DCF-DA [44]. To check the validity of the DCF-DA dye for ROS detection under our experimental conditions, we have also monitored ROS production using luminol, a method that mainly detects H_2_O_2_ [38] and could confirm ROS production after wounding (Fig. 1F). The wound sites also reacted to DAB and NBT staining confirming the formation of H_2_O_2_ and O_2_^−^ (Fig. 1C). Treatment with the NADPH oxidoreductase inhibitor DPI (Fig. 1D) or infiltration of leaves with catalase (Fig. 1E) before wounding inhibited ROS development, as measured by DCF-DA fluorescence or DAB accumulation, respectively indicating that a substantial part of ROS is O_2_^−^ and H_2_O_2_. Mutants unable to produce NO still produced ROS after wounding (Fig. 1G), making a contribution of NO to the initial wound-induced burst of ROS unlikely. Observations of plants with the LCM indicated strong fluorescence at the chloroplasts and a weaker one at the cell border after wounding (Fig. 2). The plastidic origin of some of the ROS might explain in part why ROS were still observed in *atrbohD* or *atrbohF* mutants since AtRBOHD or AtRBOHF are localized at the plasma membrane (Fig. S2). Taken together, our observations indicate a rapid (within 2 min) production of ROS after wounding (Fig. S1). Since we cannot exclude the presence of other ROS besides superoxide and H_2_O_2_, we will use the term ROS to collectively refer to the oxidative species that can be detected after wounding. Our experiments with exogenous applications of H_2_O_2_ show that ROS can have both a direct effect against *B. cinerea* and an indirect effect possibly by activation of defenses (Fig. S3). These observations are in line with previous studies showing ROS production after wounding [24], [36], [45], or in response to pathogens [26] including *B. cinerea* [46].

What is the biological importance of ROS production for WIR to *B. cinerea*?

Firstly, exogenously applied H_2_O_2_ can inhibit growth of *B. cinerea* (Fig. S3). Secondly treatments with DPI and catalase or incubation under dry conditions abolished ROS, WIR to *B. cinerea* as well as callose accumulation (Figs. 1D, E and 3). Thirdly, the absence of ROS formation and resistance to *B. cinerea* under dry conditions could be rescued in mutants impaired in ABA biosynthesis (*aba2* and *aba3*) and exogenous application of ABA suppressed both ROS and resistance to *B. cinerea* (Figs. 4 and 6C). Finally, localized treatments with cutinase resulted both in ROS production and increased resistance to *B. cinerea* on treated sites (Fig. 9) [47]. Taken together, these results support the biological importance of ROS produced in response to wounding for WIR to *B. cinerea.* Several reports have proposed that ROS and subsequent cell death formed in response to *B. cinerea* or other necrotrophs might facilitate the infection by the pathogen (reviewed in [26]). This is clearly different from the situation described here where ROS produced after wounding are strongly induced prior to an inoculation and lead to an early induction of defenses such as rapid callose formation (Figs. 1 and 3). In a previous article, we have shown wound-induced priming of camalexin synthesis, expression of *GLUTATHIONE S TRANSFERASE 1* (*GST1*) and MAPK kinase activity [34].

How do our data agree with the conventional observation that wounding is associated with susceptibility? Unless wounded plants are maintained under humid conditions WIR is lost. This resolves the apparent paradox, since most of the time plants wounded under natural conditions may not be under conditions of saturating humidity.

What is exactly the contribution of ABA? Our experiments have shown that ABA is implicated in the control of ROS formation in wounded plants that are incubated under dry conditions. Wounding followed by incubation in dry conditions lead to an increase in ABA levels (Fig. 6A). Furthermore, both the expression of ABA-dependent genes (*RAB18, RDB29* and *NCED23*) [48] and the expression of the ABA reporter gene constructs *ATH6: LUC* and *ATLTI23:LUC* [49] were induced (Figs. S4 and 6B). ABA applied on leaves suppressed wound-induced ROS and subsequent resistance to *B. cinerea* (Fig. 6C). Our data are in agreement with observations made on ABA-deficient *sitiens* mutants of tomato, where the accumulation of H_2_O_2_ was both earlier and stronger than in WT plants after inoculation with *B. cinerea* [50]. Our results and those of Asselbergh *et al*. (2007)[50] suggest a negative control of ABA on ROS formation and resistance. Several reports show a link between ABA and increased susceptibility to pathogens that was mostly explained by antagonistic interactions of ABA with defense signaling controlled by SA, JA or ET [51]. How ABA prevents wound-induced ROS accumulation in *A. thaliana* remains a study to be carried out on its own. This will be interesting, since ABA controls stomatal closure via ROS production [52]. But the action of ABA further unveiled when we observed that *aba* mutants had a resistant phenotype reminiscent of plants affected in cuticle integrity such as CUTE or the cuticle mutants *bdg* and *lacs2.3* that are immune to *B. cinerea* [32], [47]. For those reasons, ROS production was followed in *bdg or lacs2.3*. Indeed, these mutants displayed a strong DCF-DA fluorescence even after water or mock treatments or inoculation with *B. cinerea* (Fig. 5). The *bdg* or *lacs2.3* mutants were previously shown to have an increased cuticular permeability compared to WT plants (Fig. 7A, B and [32], [53]). Accordingly, we have characterized cuticular properties in *aba* mutants and observed a higher permeability in *aba2* and *aba3* mutants than in WT plants (Fig. 7A and B). Cuticular permeability measured by Calcofluor white or chlorophyll efflux was also decreased after incubation of wounded plants under dry conditions compared to plants left at high humidity despite the opening caused by the wound (Fig. 7C and D). In addition, a change was observed in the composition of aliphatic cuticle monomers [30], [31] and in the expression of the *LACS* and *BDG* genes involved in cuticle biosynthesis in wounded plants incubated under dry compared to humid conditions (Fig. 8). Incubation of wounded plants for 1.5 h under dry conditions was sufficient to increase detectable changes in of 16:0 and 18:3 cuticle monomers (Fig. S6). Thus, the changes cuticle properties in response to the environmental conditions are accompanied by changes in aliphatic monomers of the cuticle. The *aba2* and *aba3* mutants also displayed a different composition in aliphatic cuticle monomers compared to Col-0 WT plants (Fig. S6). However, it is not yet feasible to link changes in structural components of the cuticle to functions such as cuticular permeability.

Exogenous application of ABA to WT or *aba2, aba 3 lacs2.3* and *bdg* mutants decreased the cuticle permeability, further supporting a role for ABA in this process (Fig. S7). Curvers *et al*. (2010)[54] reported recently that tomato mutants impaired in ABA biosynthesis show enhanced cuticular permeability and resistance to *B. cinerea*. Our results in *A. thaliana* are in full agreement with an effect of ABA on the cuticle in tomato. It becomes now interesting to determine how ABA exerts its effects on the structure of the cuticle.

How could an increase in cuticular permeability affect the formation of ROS? The strong resistance of cuticular mutants to *B. cinerea* was explained by the facilitated diffusion of potentially antibiotic compounds produced by the plant and/or of elicitors from the medium/pathogen through the permeable cuticle surface [32], [53]. The perception of elicitors, including breakdown products of plant cuticles, has been previously described to produce ROS [22]–[24], [26]. The fact that cuticular mutants *lacs2.3*, *bdg* and *aba2*, *aba3* produced ROS even when exposed to water or mock solution alone (Fig. 5) might be explained by the perception of elicitors present at the surface of the non-sterile leaves that dissolve in the water or in the mock solution and diffuse through the cuticle to the cell where they are perceived. The hypothesis that diffusion is facilitated through a permeabilized cuticle is further supported by the effect of cutinase treatments. This enzyme was already shown to degrade cutin and increase the permeability of the cuticle and resistance to *B. cinerea* when expressed constitutively in *A. thaliana* [29], [47]. By digesting the cuticle, this enzyme generates cutin monomers, increases the permeability of the cuticle and therefore improves diffusion of breakdown products that, together with other possible elicitors present at the leaf surface, would subsequently be recognized and lead to ROS formation and resistance (Fig. 9). Recognition of cutin monomers as well as wax components has also been described to induce the production of H_2_O_2_ in abraded epicotyls of cucumber [24].

**Figure 11:**
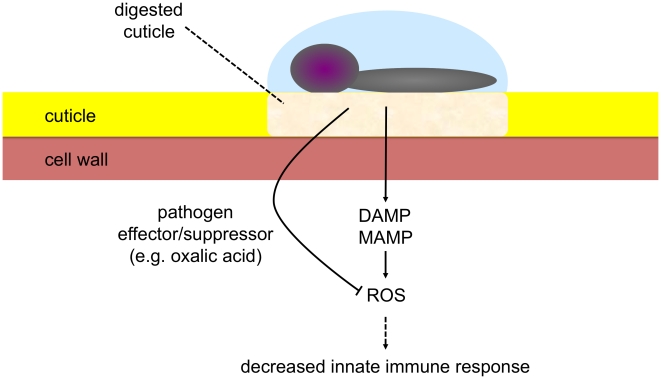
Model illustrating the role of the cuticle at the interface between *B. cinerea* and *A. thaliana*. Under the action of digestive enzymes, permeabilization of the cuticle increases, allowing for early sensing and perception of elicitors (MAMPs/DAMPs) with subsequent induction of ROS and potential activation of innate immune responses. The virulent pathogen produces effector(s)/suppressor(s) (e.g. oxalic acid) that interfere with ROS build-up leading to decreased defenses and allowing colonization.

How can these results be tied into a comprehensive model? When the cuticle is intact, it functions in protection against water loss, irradiation and xenobiotics. When it is permeabilized upon degradation by either enzymes secreted during pathogenesis or acted upon by mechanical action, elicitors might have a facilitated access to the cell, will be recognized and eventually lead to a rapid release of ROS and subsequent defense reactions. The model in Fig. 11 explains why plants are not continuously in an induced state, despite the existence of an extensive microbial flora at the leaf surface. As long as the cuticle prevents passage of elicitors, no induction of defenses takes place, illustrating economic energy management by the plant.

Why does a virulent necrotrophic pathogen like *B. cinerea* that degrades the cuticle not lead to a rapid burst of ROS? Virulent pathogens produce suppressors or effectors that can interfere with plant defenses [55]. Oxalic acid produced by *B. cinerea* [41] can suppress the formation of ROS [42] and might thus protect the fungus from their effects. We have previously shown that removal of oxalic acid produced by *B. cinerea* enhances the protection of *A. thaliana* [56]. Results obtained here with plants over expressing an oxalate decarboxylase extend these findings. In *oxdec* plants ROS appeared early during infection and growth of *B. cinerea* was decreased (Fig. 10). Similar observations were published for the virulent oxalate-producing fungus *Sclerotinia sclerotiorum* and *B. cinerea* [43], [57]. In addition, *B. cinerea* was shown to produce ABA [58], [59] that might also be possibly involved in repressing ROS production during infection. *B. cinerea* can also induce ABA production in the plant [60]. The AP-1 transcription factor Bap1 plays a pivotal role in ROS detoxification of *B. cinerea in vitro*. But Bap1 was not found to be essential for pathogenesis and the role of an oxidative burst was questioned [61]. Here we show that removal of oxalic acid can indeed restore early ROS production by the plant and impair pathogenicity of *B. cinerea*, thus confirming previous results on the importance of oxalic acid for *B. cinerea* [56], [57].

In conclusion, we propose a model whereby the cuticle is part of a sensing device besides its passive protective role of aerial plant surfaces. Without modification of the diffusive properties of the cuticle, defenses are not induced. Modifications of the surface with subsequent increased permeability will allow for a better passage for molecular determinants that can be recognized by the plant and lead to the activation of defenses. This mechanism is doubled up by another mechanism provided by the wall itself, that, when exposed to the appropriate enzymes will breakdown to damage associated determinants (DAMPs) that are also recognized and initiate defenses. Virulent pathogens have evolved mechanisms to interfere with such mechanisms and data presented here support these findings. Future work should now be directed at the molecular mechanisms that lead to a rapid generation of ROS. It will be interesting to determine if they overlap with a similar responses observed when plant react to other elicitors that lead to ROS formation such as flagellin.

## Materials and Methods

### Plant maintenance

*Arabidopsis thaliana* seeds were grown on a pasteurized soil mix of humus and Perlite (3∶1). Seeds were kept at 4°C for two days and then transferred to the growth chamber. Plants were grown in a 12 h light/12 h dark cycle with 60–70% of relative humidity, with a day temperature of 20–22°C and a night temperature of 16–18°C. WT plants were obtained from the Nottingham *Arabidopsis* Stock Center (Nottingham, UK). The Arabidopsis mutant referred to as *aba2* was *aba2–1* and *aba3* was *aba3–1* [62]. The *lacs2–3, bdg2* and the *nia1nia2noa1–2* mutants were previously described [31], [32], [39].

### Culture of *B. cinerea*, inoculation, staining of hyphae and wounding procedure

*B. cinerea* strains BMM, provided by Brigitte Mauch-Mani (University of Neuchâtel, Switzerland), were grown on Difco (Becton Dickinson, http://www.bd.com) potato dextrose agar 39 g l^−1^. Spores were harvested in water and filtered through glass wool to remove hyphae. Spores were diluted in ¼ strength Difco potato dextrose broth (PDB) at 6 g l^−1^ for inoculation. Droplets of 6 µl spore suspension (5×10^4^ spores ml^−1^) were deposited on leaves of 4-week-old plants for quantification of lesions size (mm) after 3 days. Spores (2×10^5^ spores ml^−1^) were also sprayed on whole plants for RT-PCR experiments. The inoculated plants were kept under high humidity in covered trays. Control plants were mock inoculated with ¼ strength PDB solution. Leaves were wounded by gently pressing the lamina with a laboratory forceps. For wounding of entire leaves, the pressing was carried out on both sides of the main vein. Wounded leaves were incubated in covered trays at high humidity (referred to as humid conditions); in some cases the trays were left uncovered after wounding (referred to as dry conditions) under the same laboratory conditions. Inoculation with *B. cinerea* was performed within 10 min after wounding, by placing a droplet of spores on the wound site. Fungal structures and dead plant cells were stained by boiling inoculated leaves for 5 min in a solution of alcoholic lactophenol trypan blue. Stained leaves were extensively cleared in chloral hydrate (2.5 g ml^−1^) at room temperature by gentle shaking, and then observed using a Leica DMR microscope with bright-field settings.

### Detection of ROS

ROS were detected using the fluorescent probe 5-(and 6)-carboxy-2′,7′-dichloro dihydrofluorescein diacetate (DCF-DA) (Sigma-Aldrich, www.sigmaaldrich.com). Wounded or unwounded leaves were vacuum-infiltrated (3×3 min) in 60 µM of DCF-DA in a standard medium (1 mM KCl, 1 mM MgCl_2_, 1 mM CaCl_2_, 5 mM 2-morpholinoethanesulfonic acid adjusted to pH 6.1 with NaOH) [44]. Leaves were then rapidly rinsed in DCF-DA medium and observed using a Leica DMR epifluorescence microscope with a GFP filter set (excitation 480/40 nm, emission 527/30 nm) (Leica, www.leica.com). Microscope images were saved as TIFF files and processed for densitometric quantification with Image J version 1.44 (NIH). Software settings were kept the same for every image analyzed; the surface of each analyzed picture was the same (2.278 mm^2^). One representative image of the fluorescent leaf surface was placed above each histogram as a visual illustration. Quantification of ROS using DCF-DA was also performed on wounded or unwounded leaf discs of 5 mm incubated in 60 µM of DCF-DA in a 96-well plate (Sarstedt, www.sarstedt.com). After vacuum-infiltration (3×3 min), ROS were determined using a FL×800 microplate fluorescence reader with a excitation filter 485/20 nm and an emission filter 528/40 nm (Bio-Tek instruments, www.biotek.com). Accumulation of O_2_^−^ and H_2_O_2_ in leaves was determined using nitroblue tetrazolium (NBT) staining [63] and 3,3′-diaminobenzidine (DAB) staining [64] respectively. The destained leaves were observed using a Leica DMR microscope with bright-field settings. The H_2_O_2_ accumulation was also determined using the luminol test [65]. A solution containing 50 µl of 0.5 mM luminol (3-aminophtalhydrazide, Sigma-Aldrich) in 0.2 N NH_3_, pH 9.5 added to 0.8 ml of 0.2 N NH_3_, pH 9.5 and 100 µl of 0.5 mM K_3_Fe(CN)_6_ in 0.2 N NH_3_, pH 9.5 was added to leaf discs of 8 mm in 24-well plates (Corning incorporated, www.corning.com) and luminescence was measured immediately using CCD camera (Princeton Instrument Versarray system, www.princetoninstruments.com) equipped with a Sigma Aspherical objective (www.sigma-foto.de) in a dark box. The pictures were analyzed with an Imaging System.

### Luciferase activity

Arabidopsis reporter lines consisting of either a *pAtHB6* or *pLTI65* promoter fragment fused to the *LUC* gene were generously given by Prof. Erwin Grill [66]. For imaging of LUC activity, plants were sprayed with a solution of 1 mM luciferin (Applichem, www.applichem.com) in 10 mM MES, pH 7.0, 0.01% Tween 80. Ten min after luciferin spraying, light emission was detected using an intensified CCD camera (Princeton Instrument Versarray system, www.princetoninstruments.com) equipped with a Sigma Aspherical objective (www.sigma-foto.de) in a dark box. The pictures were analyzed with the MetaVue Imaging System (www.biovis.com/metavue.htm).

### Callose staining

Twenty-four hours post infiltration, leaves were harvested and distained in 3∶1 ethanol: lactic acid, previously diluted in 1:2 ethanol. The solution was changed several times until the chlorophyll had totally disappeared. Translucent leaves were progressively re-hydrated in 70% ethanol for about 2 hours and in 50% ethanol for 2 hours. Leaves were left in water and gently shaken overnight. Leaves were then incubated for 24 hours in 150 mM K_2_HPO_4_ (pH 9.5) containing 0.01% aniline blue. Stained material was mounted on glass slides in 50% glycerol and examined under UV light with a LEICA DMR fluorescence microscope. The callose deposition was determined by counting the pixels using the Image J 1.44 software (NIH).

### RNA extraction and real time RT-PCR

RNA was prepared using the Trizol reagent containing 38% saturated phenol, 0.8 M guanidine thiocyanate, 0.4 M ammonium thiocyanate, 0.1 M sodium acetate and 5% glycerol. RNA (1 µg) was then retrotranscribed into cDNA (Omniscript RT kit, Qiagen, www.qiagen.com). RT-PCR was performed using Sensimix SYBR Green Kit (Bioline, www.bioline.com). Gene expression values were normalized to expression of the plant gene At4g26410, previously described as a stable reference gene [67]. The primers used were rab18fw 5′- AACATGGCGTCTTACCAGAA; rab18rev 5′-AGTTCCAAAGCCTTCAGTCC; rd29bfw 5′-GAATCAAAAGCTGGGATGGA; rd29brev 5′-TGCTCTGTGTAGGTGCTTGG; nced23fw 5′-ATTGGCTATGTCGGAGGATG; nced23rev 5′-CGACGTCCGGTGATTTAGTT; lacs2-3fw 5′-GTGCCGAGAGGAGAGATTTG; lacs2-3rev 5′-CGAGGTTTTCAACAGCAACA; bdgfw 5′-TTCTTGGCTTTCCTCTTCCA; bdgrev 5′- CCATAACCCAACAGGTCCAC.

### Treatments with ABA, DPI, catalase and cutinase

Leaf discs of 8 mm were floated in 24-well plates (Corning incorporated, www.corning.com) filled with 1.5 ml in each well of either 50 µM DPI (Sigma-Aldrich), 100 µM ABA (Sigma-Aldrich) in 0.05% EtOH and distilled water or EtOH 0.05% as controls for 24 h before wounding. Catalase (300 U ml^−1^ catalase; Sigma-Aldrich) was infiltrated into the leaves prior to wounding. After wounding, either ROS were visualized on the discs with DCF-DA or DAB or discs were floated on distilled water for 24 h and inoculated with *B. cinerea* and infection was determined after 3 days. The effect of ABA on cuticle permeability was determined after spraying leaves with 100 µM ABA in 0.05% EtOH followed by a 24 h incubation period under humid conditions. Eight mL droplets of purified preparation of cutinase (5 mg l^−1^) from *F. oxysporum* [68] or 10 mM Na-acetate pH 5.2 in controls were applied on the leaf surface. After 72 h incubation under moist conditions, the droplets were removed and leaves were stained with DCF-DA or DAB to detect ROS. Alternatively, the droplet was rinsed off the leaf and replaced by a droplet of spore suspension of *B. cinerea* and incubated as described above.

### Quantification of abscisic acid

Leaf material (ca 500 mg) was frozen in liquid nitrogen and collected in 2 ml Eppendorf tubes, and then homogenized (twice) with 1000 µl of 70°C-warm extraction buffer (water/propane-1-ol/HCl : 1/2/0.005, v/v) without thawing. The sample was transferred to a glass tube and 200 ng of the internal standard abscisic acid-d6 (Santa Cruz Biotechnology, www.scbt.com) and 2 ml of dichloromethane were added. The sample was then mixed 15 s with a vortex and centrifuged 1 min at 14,000 g. The lower organic phase was transferred to a new glass tube and dried by the addition of anhydrous Na_2_SO_4_. Then, carboxylic acids including ABA were methylated to their corresponding methyl esters at room temperature for 30 min after the addition of 10 µl of 2 M bis-trimethylsilyldiazomethane (Sigma-Aldrich) and 100 µl of MeOH. Methylation was stopped by the addition of 10 µl of 2 M acetic acid during 30 min at room temperature. Extraction of the vapor phase was performed using a VOC column conditioned with 3×1 ml of dichlororomethane. The VOC column and a nitrogen needle were fixed on the screw cap of the tube. The solvent was evaporated under a nitrogen stream at 70°C and heated for 2 min at 200°C. The VOC column was eluted with 1 ml of dichloromethane in a new glass tube. The eluate was evaporated and then dissolved in 20 µl of hexane before injecting 3 µl on a capillary column HP1 (25 m×0.25 mm) GC column (Agilent, www.agilent.com) fitted to a Hewlett Packard 5980 GC coupled to a 5970 mass specific detector. The methyl esters of ABA and ABA-d6 were detected and quantified by selective ion monitoring at m/z 190 and 194 respectively. The amount of ABA (measured as methyl ABA) was calculated by reference to the amount of internal standard. The results are expressed in ng mg^−1^ fresh weight of plant tissue.

### Chemical composition of cuticle

The surface of 15 to 20 leaves of 4 weeks-old *A. thaliana* Col-0 was first determined as described previously [69]. Then the leaves were extracted with chloroform:methanol (1∶1; v/v) and dried before depolymerization. The samples were depolymerized using transesterification with 2 ml BF_3_ (Fluka, Sigma-Aldrich) for 12 h at 75°C. After addition of 2 ml saturated NaCl/H_2_O and 20 µg dotriacontane as internal standard, aliphatic monomers were extracted 3 times with 1 ml of chloroform. The combined organic phase was evaporated in a stream of nitrogen to a volume of ∼100 µl. All samples were treated with bis-(*N*,*N*,-trimethylsilyl)-tri-fluoroacetamide (BSTFA; Macherey-Nagel, www.mn-net.com) for 40 min at 70°C to convert free hydroxyl and carboxyl groups into their corresponding trimethylsilyl (TMS) derivatives. Remaining solvent and derivatization reagents were removed under a stream of N_2_ and the samples were resolubilized in 100 ml dichloromethane prior to vapour phase extraction. Monomers were identified on the basis of their electron-impact MS spectra (70 eV, m/z 50–700) on a HP 6890 GC system coupled to an HP 5973 mass-selective detector (USA). The depolymerisation products were separated by on a capillary column (ZB-AAA, 10 m, 0.25 mm, Zebron, Phenomenex, www.phenomenex.com) by injection at 50°C, 2 min at 50°C, 5 °C min^−1^ to 225°C, 1 min at 225°C, 20°C min^−1^ to 310°C, 10 min at 310°C. The results are expressed in g cm^−2^ leaf tissue.

### Tests of cuticle permeability

Chlorophyll extraction and quantification was performed according to the protocol of Sieber *et al*. 2009 [29]. Leaves were cut at the petiole, weighed and immersed in 30 ml of 80% ethanol. Chlorophyll was extracted in the dark at room temperature with gentle agitation. Aliquots were removed at 2, 5, 10, 20, 30 and 40 min after immersion. After ABA treatment, aliquots were removed at 40 and 60 min after immersion in ethanol. The chlorophyll content was determined by measuring absorbance at 664 and 647 nm and the micromolar concentration of total chlorophyll per gram of fresh weight of tissue was calculated from the following equation: (7.93×(*A*_664_ nm) + 19.53×(*A*_647 _nm)) g^−1^ fresh weight. The toluidine blue test was carried out by placing 6 ml droplets of a 0.025% toluidine blue solution in ¼ PDB placed on the leaf surface. After 2 h incubation leaves were washed gently with distilled water to remove excess of the toluidine blue solution from leaves. For staining with Calcofluor white, leaves were bleached in absolute ethanol overnight, equilibrated in 0.2 M NaPO_4_ (pH 9) for 1 h, and incubated for 1 min in 0.5% Calcofluor white in 0.2 M NaPO_4_ (pH 9). Leaves were rinsed in NaPO_4_ buffer to remove excess of Calcofluor white and viewed under UV light on a GelDoc 2000 system (Biorad, www.biorad.com).

### Production of *oxdec* lines and detection of the oxalic acid decarboxylase activity

The gene used to transform *A. thaliana* plants is the *OXALATE DECARBOXYLASE* from *Trametes versicolor* (TOXDEC, Genbank accession number AY370675). The gene was kindly provided by Andreas Walz (Institute for Phytomedicine, University of Hohenheim) as cDNA cloned into the transformation vector pBI 101 (p221-TOXDC). *A*. *thaliana* Col-0 plants were transformed using *Agrobacterium tumefaciens* and the flower dip method [70]. Resulting seeds were collected and grown on selective medium; the expression of the gene was determined respectively by crude PCR and Northern Blot. The activity of oxalate decarboxylase was measured in the T3 generation along with resistance to *B. cinerea* strain BMM. Oxalate decarboxylase activity was measured as described [71] with the following modifications. Leaves (100 mg) were homogenized using a Polytron (Kinematica, www.kinematica-inc.com) in 1 ml extraction buffer containing 50 mM potassium phosphate buffer pH 7.5, 1 mM EDTA, 1 mM phenylmethylsulphonyl fluoride and 5 mM sodium ascorbate; 200 µl of this extract was added to 10 µl oxalic acid (1 M, pH 6.2) and incubated 3 h at 37°C. After centrifugation during 5 min, 12 µl of a 30% sodium acetate solution (w/v) and 300 µl reagent solution containing 0.5 g citric acid monohydrate, 10 g acetic acetamide in 100 ml isopropanol were added to 150 µl extract. The solution added to 1 ml of acetic acid anhydride was incubated 40 min at 50°C; the intensity of the resulting pink color reflects oxalate decarboxylase activity.

### Statistical analyses

Kruskal-Wallis one way analysis of variance (ANOVA) on ranks followed by a Dunn's test was performed using SigmaPlot version 11.1 software (Systat Software, San Jose, CA). Different letters above each bar represent statistically significant differences (Dunn's test; P<0.05).

## Supporting Information

Figure S1 **Time-course of ROS after wounding.** To follow the rapid formation of ROS (measured as DCF-DA fluorescence), WT leaves were infiltrated with DCF-DA and then wounded. The first fluorescent signal was detected 2 min after wounding. The experiment was repeated twice with similar results.
(TIF)

Figure S2 **ROS production in** ***NADPH oxidase*** **mutants.** ROS (measured as DCF-DA fluorescence) and WIR to *B. cinerea* were still detected after wounding in *atrboh D* and *atrboh F* as well as in the double mutant *atrboh D/F.* After wounding, all plants were kept under humid conditions. W: wounded; Ctrl: unwounded control plants. The experiment was carried out twice with similar results. Different letters above each bar represent statistically significant differences (Dunn's test; P<0.05).
(TIF)

Figure S3 **Direct and indirect effects of H_2_O_2_ against** ***B. cinerea*** **.** (A) Leaves were treated with H_2_O_2_ or water (Ctrl) (during 1 d in high humidity) then rinsed with water and subsequently inoculated with *B. cinerea* (n = 15; ±SD). Different letters above each bar represent statistically significant differences (Dunn's test; P<0.05). (B) Effect of H_2_O_2_ or water (Ctrl) on *in vitro* hyphal growth of *B. cinerea* (observed 16 h after treatment). The experiment was carried out twice times with similar results.
(TIF)

Figure S4 **Expression of ABA-dependent genes** ***RAB18, RD298*** **and** ***NCED23*** **.** Leaves were wounded and maintained for 1.5 h under high humidity in tightly covered well-watered trays (humid) or in uncovered trays at room conditions (dry) prior to expression of ABA-dependent genes. Gene expression was determined 0, 15 or 30 min after wounding in plants incubated under humid or dry conditions and either mock-inoculated (M) or inoculated with *B. cinerea* (*Bc*) (n = 3; ±SD). The experiment was carried out twice with similar results
(TIF)

Figure S5 **Number of stomata in** ***lacs2.3, bdg, aba2 and aba3*** **mutants compared to WT plants (n = 10; ±SD).** Different letters above each bar represent statistically significant differences (Dunn's test; P<0.05). The experiment was carried out twice with similar results.
(TIF)

Figure S6 **Composition of aliphatic monomers of** ***A. thaliana*** **and ABA mutants leaf cuticle.** Wild type (WT) leaves were wounded and maintained for 1.5 h under high humidity in tightly covered well-watered trays (humid) or in uncovered trays at room conditions (dry) prior to fatty acid analysis. The fatty acid composition was determined for 15 to 20 leaves of wounded and unwounded WT plants in dry and humid conditions and of *aba* mutants (n = 3; ±SD). D: dry; H: humid; W: wounded. For each fatty acid, different letters above each bar represent statistically significant differences (Dunn's test; P<0.05).
(TIF)

Figure S7 **Cuticle permeability is impaired in WT, ABA mutants and cuticle mutants after ABA treatment.** Chlorophyll leaching decreased upon ABA treatment (+ABA) in WT, *aba2, aba3, lacs2.3* and *bdg* mutants compared to untreated plants (-ABA) (measured at 40 and 60 min after immersion in ethanol 80%). Plants were treated with ABA 100 mM for 24 h under humid conditions (n = 4; ±SD). Different letters above each bar represent statistically significant differences (Dunn's test; P<0.05).
(TIF)
