# Supplementary figures and images for: A Permeable Cuticle Is Associated with the Release of Reactive Oxygen Species and Induction of Innate Immunity
Source: PLoS Pathog. 2011 Jul 28;7(7):e1002148. doi: 10.1371/journal.ppat.1002148 (PMC3145797; doi:10.1371/journal.ppat.1002148)

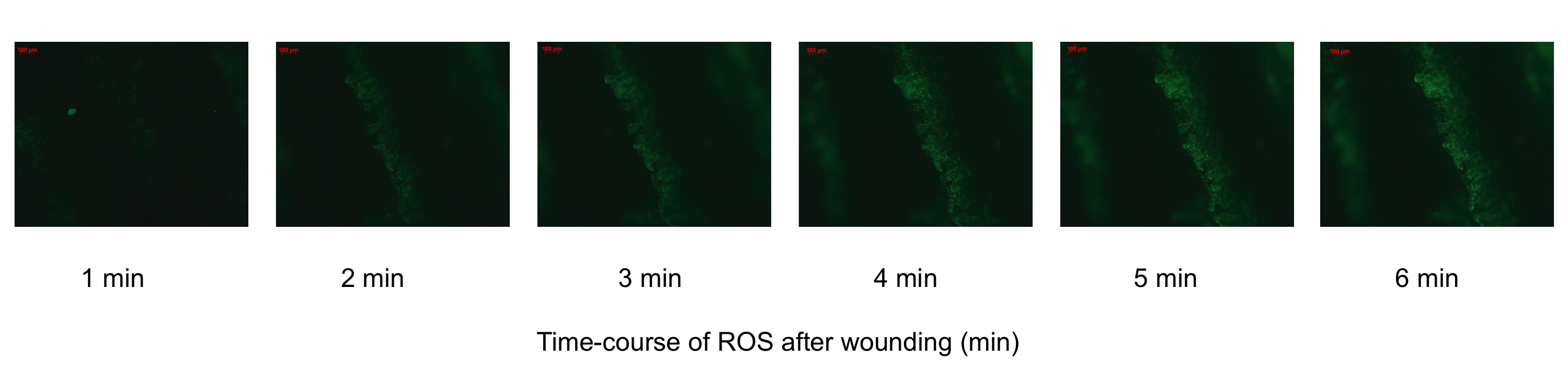

Supplement: Figure S1 — Time-course of ROS after wounding. To follow the rapid formation of ROS (measured as DCF-DA fluorescence), WT leaves were infiltrated with DCF-DA and then wounded. The first fluorescent signal was detected 2 min after wounding. The experiment was repeated twice with similar results. (TIF) [file ppat.1002148.s001.tif]

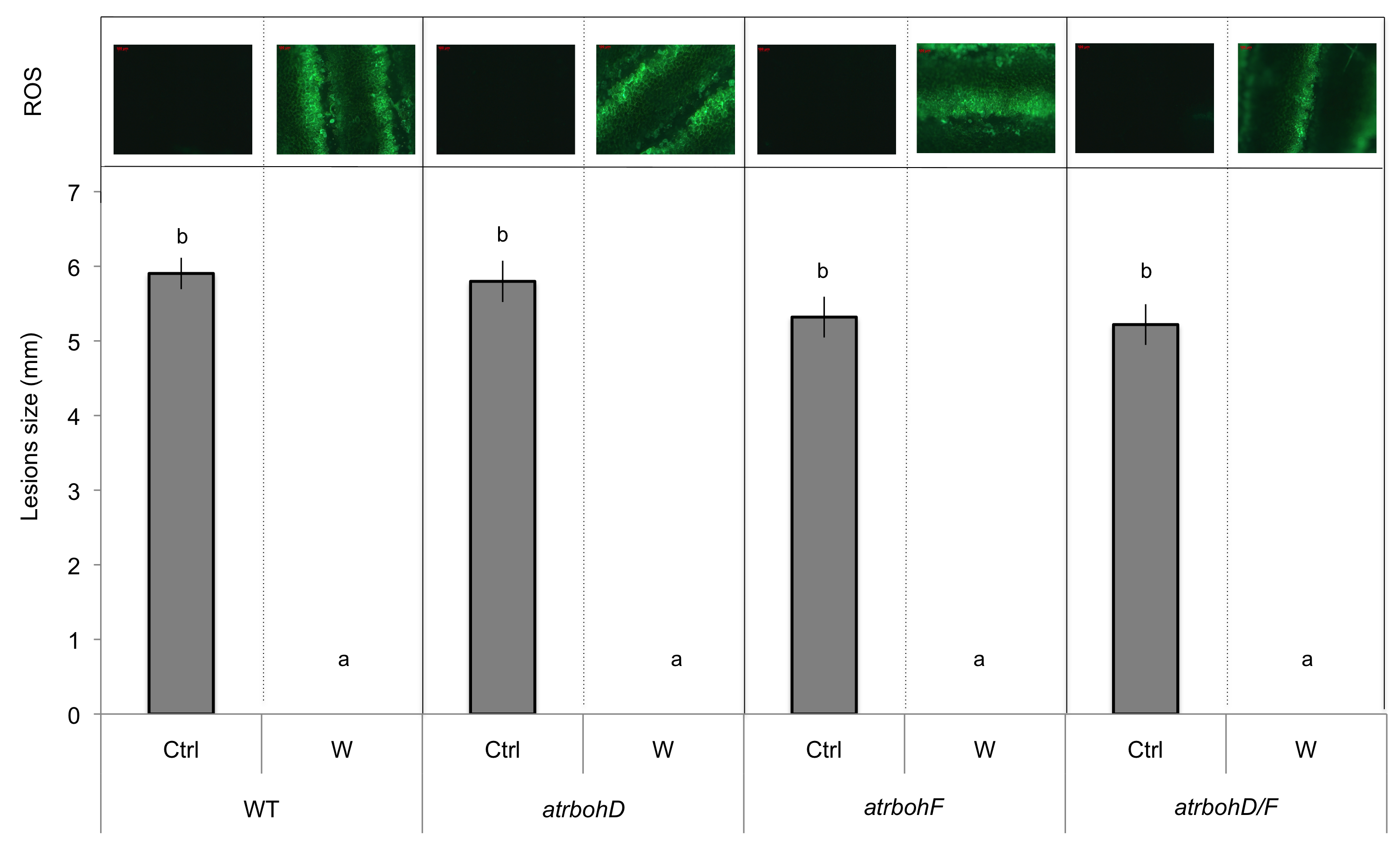

Supplement: Figure S2 — ROS production in NADPH oxidase mutants. ROS (measured as DCF-DA fluorescence) and WIR to B. cinerea were still detected after wounding in atrboh D and atrboh F as well as in the double mutant atrboh D/F. After wounding, all plants were kept under humid conditions. W: wounded; Ctrl: unwounded control plants. The experiment was carried out twice with similar results. Different letters above each bar represent statistically significant differences (Dunn's test; P<0.05). (TIF) [file ppat.1002148.s002.tif]

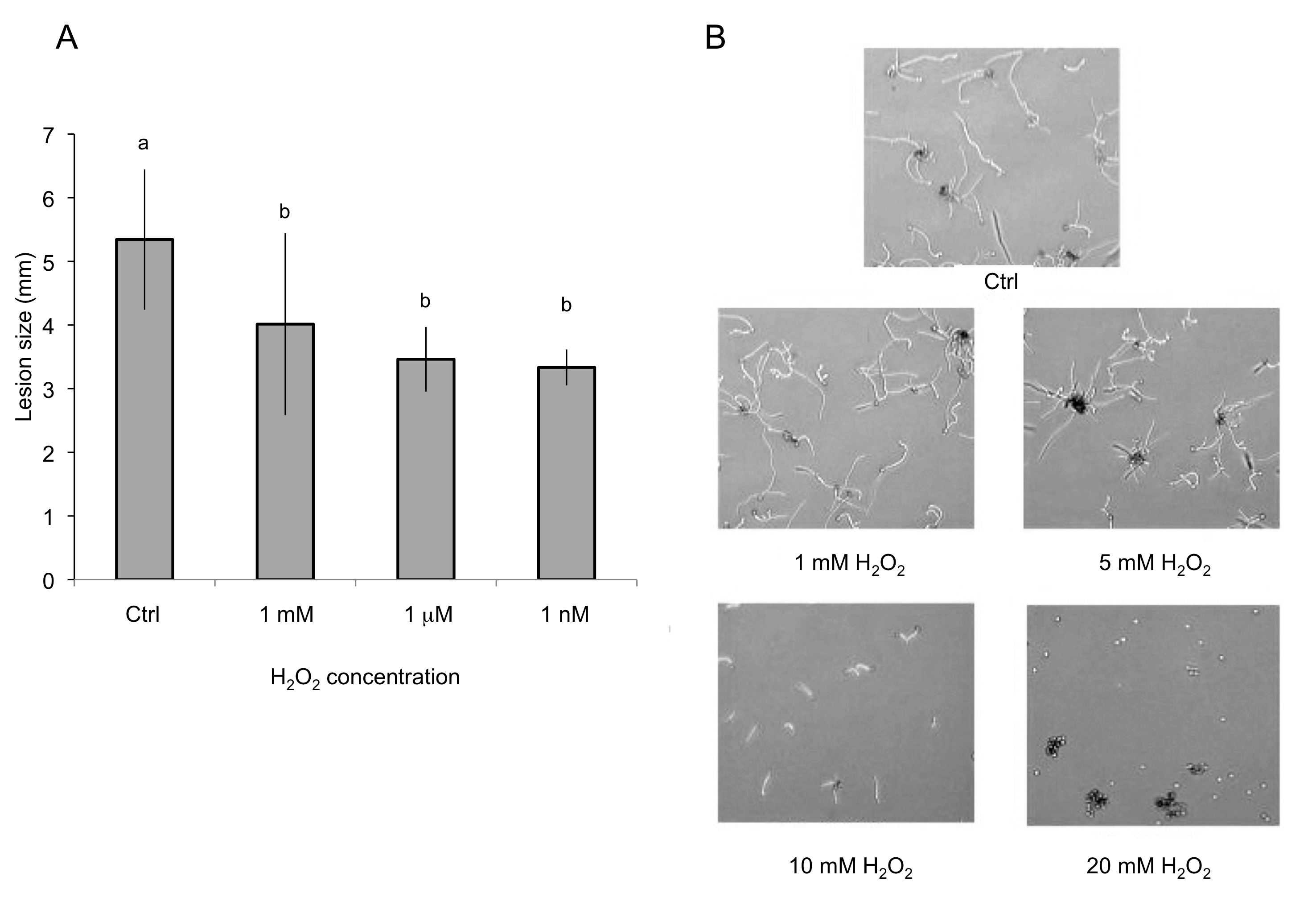

Supplement: Figure S3 — Direct and indirect effects of H2O2 against B. cinerea . (A) Leaves were treated with H2O2 or water (Ctrl) (during 1 d in high humidity) then rinsed with water and subsequently inoculated with B. cinerea (n = 15; ±SD). Different letters above each bar represent statistically significant differences (Dunn's test; P<0.05). (B) Effect of H2O2 or water (Ctrl) on in vitro hyphal growth of B. cinerea (observed 16 h after treatment). The experiment was carried out twice times with similar results. (TIF) [file ppat.1002148.s003.tif]

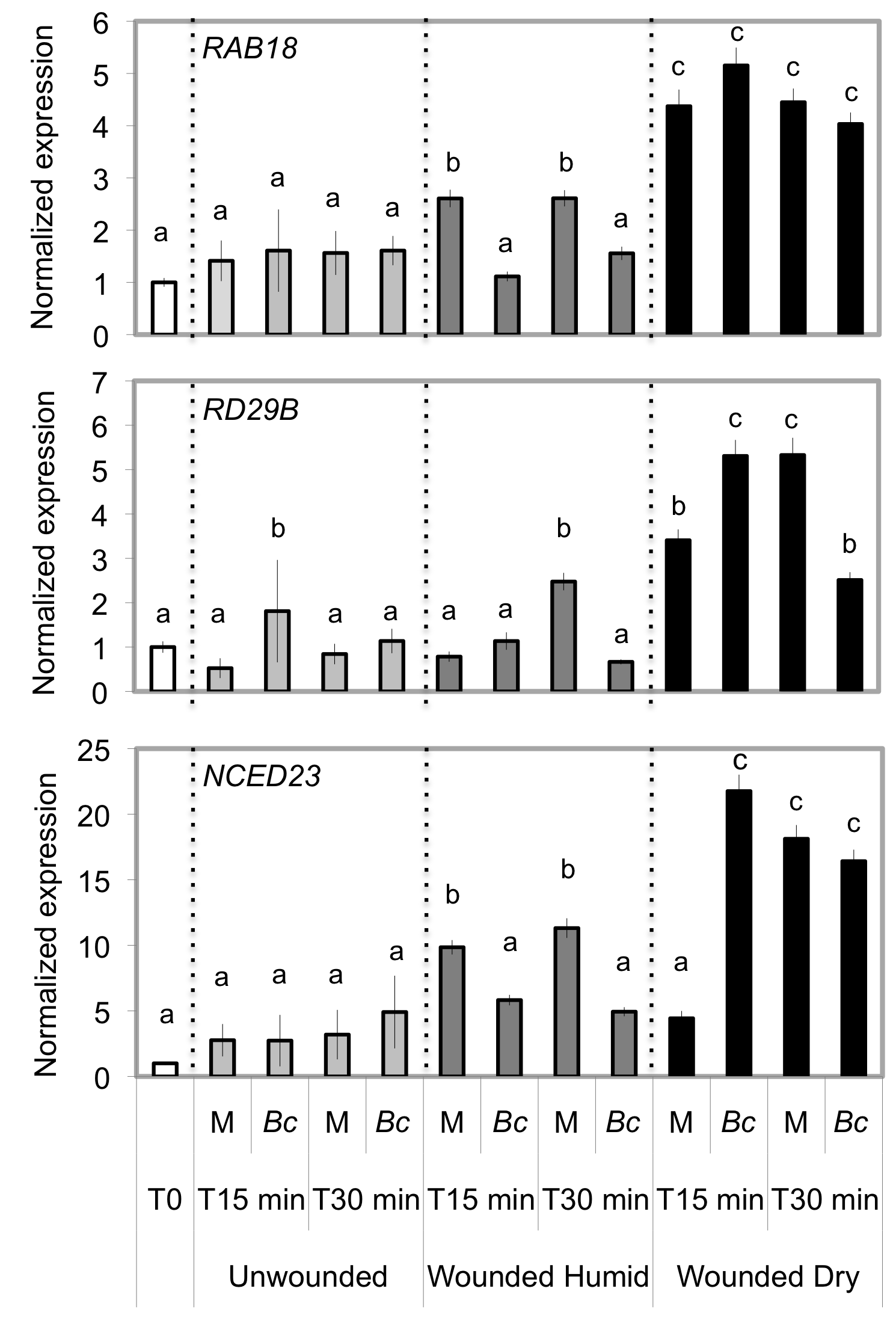

Supplement: Figure S4 — Expression of ABA-dependent genes RAB18, RD298 and NCED23 . Leaves were wounded and maintained for 1.5 h under high humidity in tightly covered well-watered trays (humid) or in uncovered trays at room conditions (dry) prior to expression of ABA-dependent genes. Gene expression was determined 0, 15 or 30 min after wounding in plants incubated under humid or dry conditions and either mock-inoculated (M) or inoculated with B. cinerea (Bc) (n = 3; ±SD). The experiment was carried out twice with similar results (TIF) [file ppat.1002148.s004.tif]

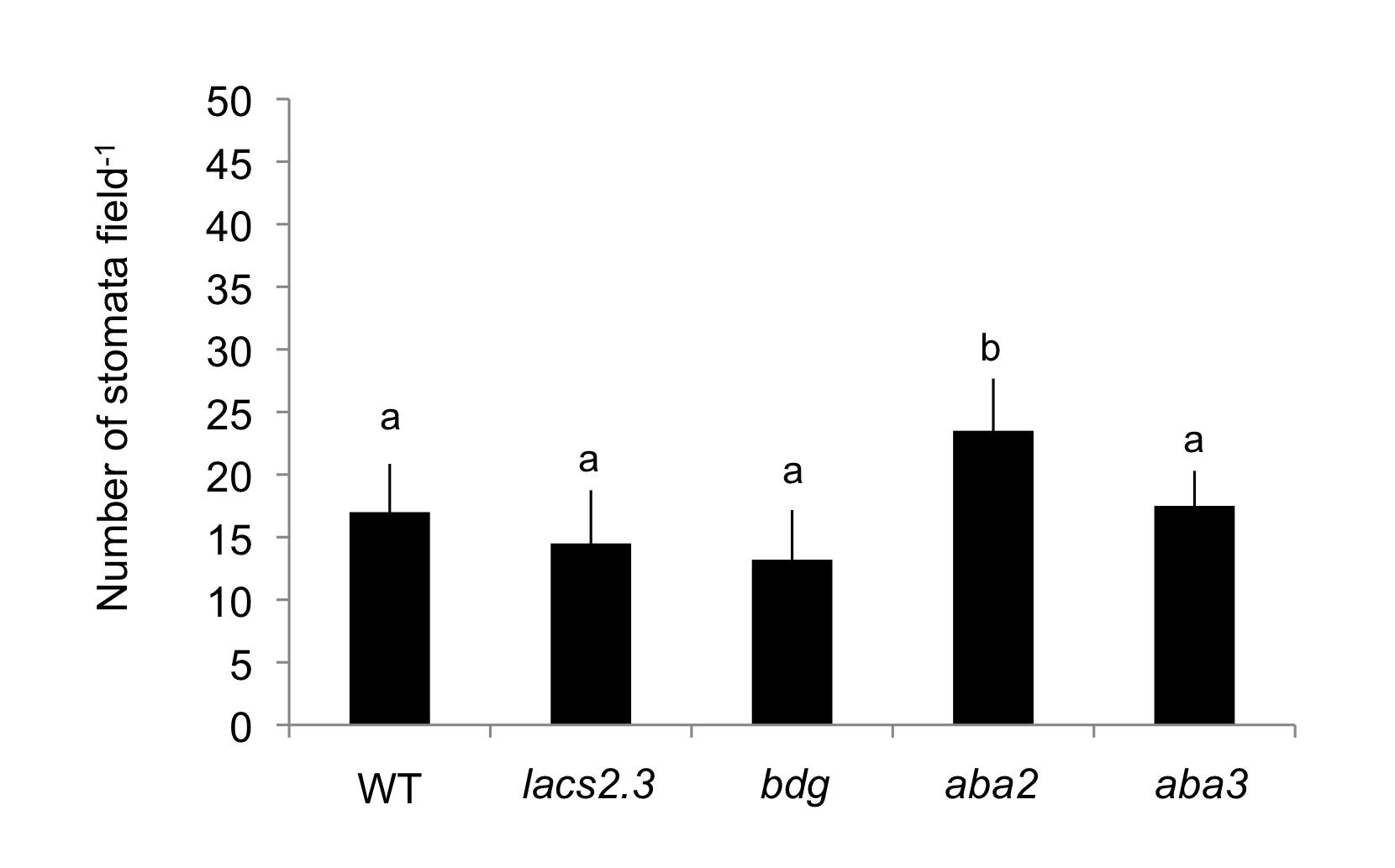

Supplement: Figure S5 — Number of stomata in lacs2.3, bdg, aba2 and aba3 mutants compared to WT plants (n = 10; ±SD). Different letters above each bar represent statistically significant differences (Dunn's test; P<0.05). The experiment was carried out twice with similar results. (TIF) [file ppat.1002148.s005.tif]

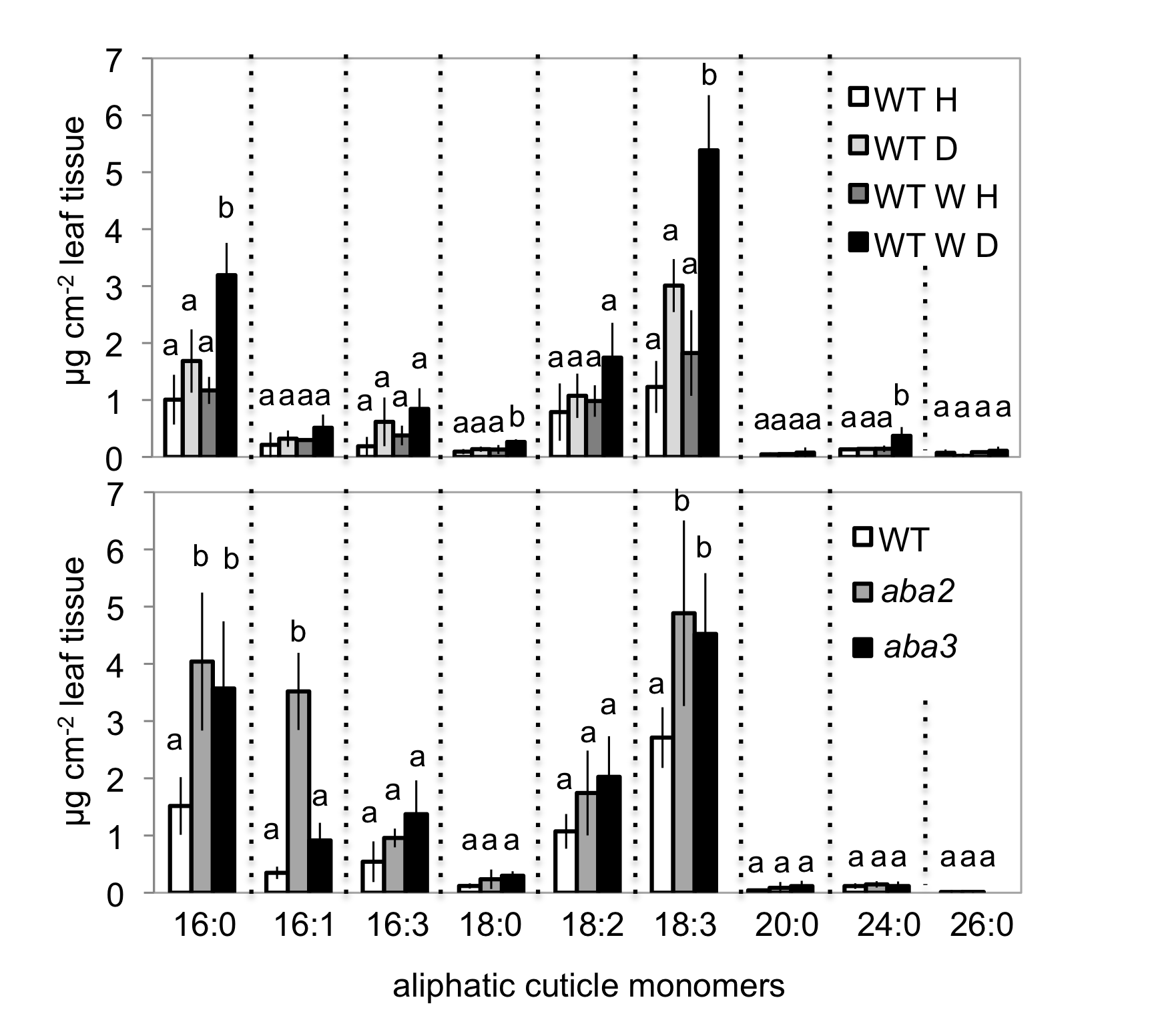

Supplement: Figure S6 — Composition of aliphatic monomers of A. thaliana and ABA mutants leaf cuticle. Wild type (WT) leaves were wounded and maintained for 1.5 h under high humidity in tightly covered well-watered trays (humid) or in uncovered trays at room conditions (dry) prior to fatty acid analysis. The fatty acid composition was determined for 15 to 20 leaves of wounded and unwounded WT plants in dry and humid conditions and of aba mutants (n = 3; ±SD). D: dry; H: humid; W: wounded. For each fatty acid, different letters above each bar represent statistically significant differences (Dunn's test; P<0.05). (TIF) [file ppat.1002148.s006.tif]

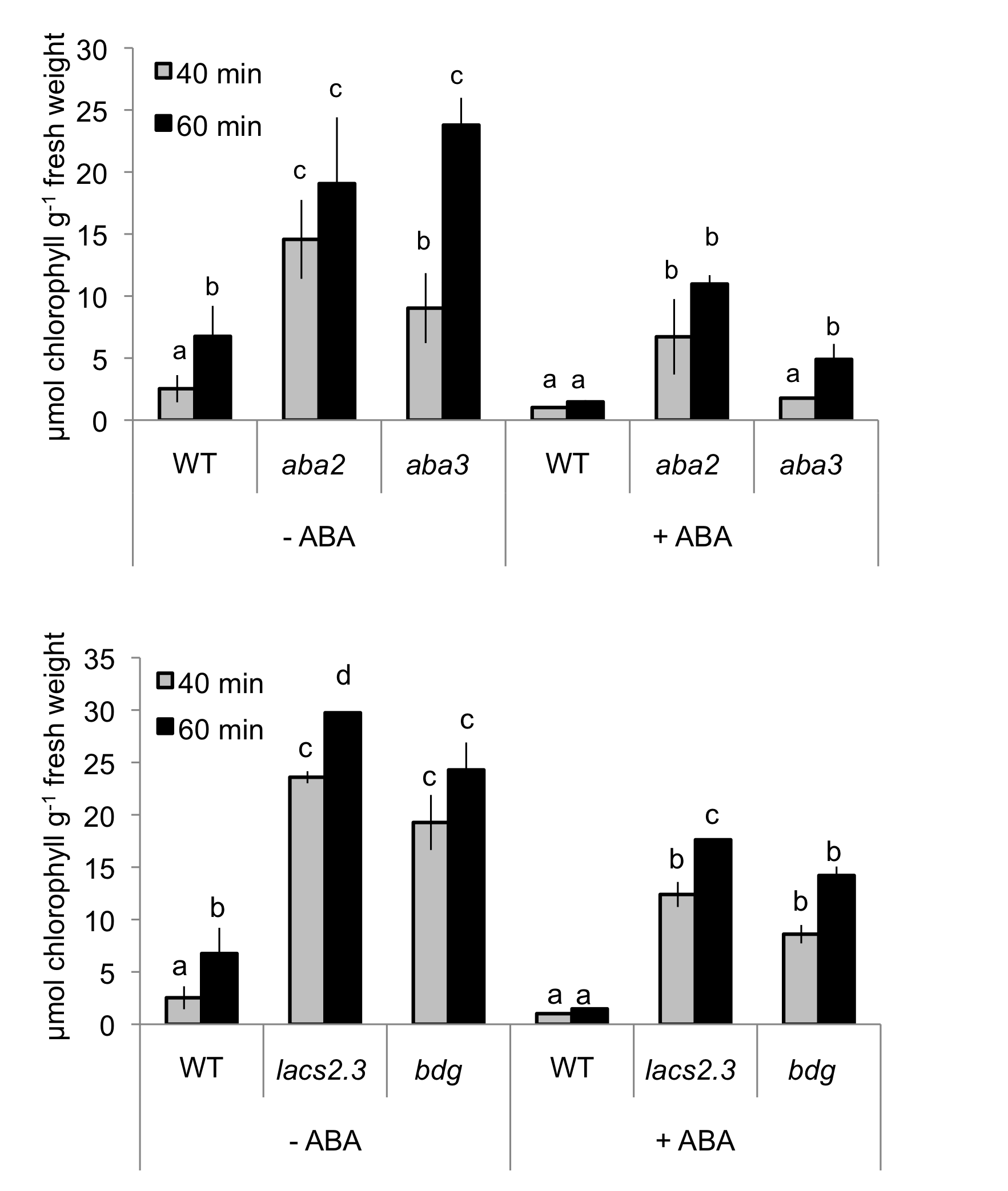

Supplement: Figure S7 — Cuticle permeability is impaired in WT, ABA mutants and cuticle mutants after ABA treatment. Chlorophyll leaching decreased upon ABA treatment (+ABA) in WT, aba2, aba3, lacs2.3 and bdg mutants compared to untreated plants (-ABA) (measured at 40 and 60 min after immersion in ethanol 80%). Plants were treated with ABA 100 mM for 24 h under humid conditions (n = 4; ±SD). Different letters above each bar represent statistically significant differences (Dunn's test; P<0.05). (TIF) [file ppat.1002148.s007.tif]
